# Alternative bait trials in the Barents Sea snow crab fishery

**DOI:** 10.7717/peerj.6874

**Published:** 2019-05-13

**Authors:** Tomas Araya-Schmidt, Leonore Olsen, Lasse Rindahl, Roger B. Larsen, Paul D. Winger

**Affiliations:** 1 Fisheries and Marine Institute, Memorial University of Newfoundland, St. John’s, NL, Canada; 2 Escuela de Ciencias del Mar, Facultad de Ciencias del Mar y Geografía, Pontificia Universidad Católica de Valparaíso, Valparaíso, Chile; 3 SINTEF Nord AS, Tromsø, Norway; 4 The Arctic University of Norway UIT, Tromsø, Norway

**Keywords:** Traps, Snow crab, Barents Sea, Seal, Whale, Squid, Alternative baits, Bait, Norway

## Abstract

Commercial harvesting of snow crab (*Chionoecetes opilio*) in the Barents Sea started in 2012 by Norwegian fishing vessels. This new fishery has significant bait requirements, representing an emerging conservation challenge. In this study, we evaluate the performance of five alternative (natural) baits manufactured from the waste stream of existing and sustainably managed harp seal (*Pagophilus groenlandicus*) and minke whale (*Balaenoptera acutorostrata*) capture. Five different types of new bait were evaluated, including seal fat (SF), seal fat with skin (SFS), seal meat with bone (SMB), whale fat with skin (WFS), and whale meat with fat (WMF). A comparative fishing experiment was conducted onboard a commercial snow crab fishing vessel in the Barents Sea (May–June, 2016) to evaluate the performance of traditional bait (squid, *Illexs spp*.) and alternative baits at catching snow crabs. Performance of the different baits were compared on the basis of the number of commercial crab caught per trap haul catch per unit effort (CPUE) and carapace width (CW). Our results showed that SF and SFS performed equally well to traditional bait, with no statistical difference in CPUE (*p*-value = 0.325 and 0.069, respectively). All of the other experimental baits significantly decreased CPUE, when compared to squid. No significant effect of bait treatment on CW was detected and the cumulative distribution of CW was the same between control traps and each of the bait treatments. Overall the results indicated that SF and SFS represent a viable alternative to replace traditional bait, addressing a key conservation challenge in this bait intensive snow crab fishery.

## Introduction

Snow crab (*Chionoecetes opilio*) is considered an invasive species in the Barents Sea. It is unknown how or when this species populated the Barents Sea (Hansen, 2016). Since the first findings in the southeastern part of the Barents Sea (Kuzmin, Akhtarin & Menis, 1988), the abundance and distribution of snow crab has increased steadily every year (Alvsvåg, Agnalt & Jørstad, 2009; Pavlov & Sundet, 2011). Snow crab colonized favorable conditions in the Barents Sea, including depths, substrates, and temperature ranges that match its biological preferences. It is currently distributed in areas with bottom temperatures ranging from −0.7 to 3.4 °C and at depths between 180 and 350 m (Alvsvåg, Agnalt & Jørstad, 2009). Its preferred habitat is currently found in the northern parts of the Russian Exclusive Economic Zone and in international waters of the Barents Sea, covering an overall area more than 34% of the Barents Sea (Kaiser, Kourantidou & Fernandez, 2018).

Commercial harvesting of snow crab in the Barents Sea started in 2012 by Norwegian vessels (Norges Råfisklag, 2019). Norwegian landings in 2012 were 2.5 tonnes and increased rapidly reaching up to 5,405 tonnes in 2016, to then decrease to 3,067 and 2,805 tonnes in 2017 and 2018, respectively (Norges Råfisklag, 2019). Total landed value peaked in 2016 at 192 million NOK (∼$22.6 million USD) and decreased to 165 million NOK in 2018 (∼$19.3 million USD) (Norges Råfisklag, 2019).

This new fishery requires substantial amounts of natural bait to attract snow crabs into the traps (one kg of bait per trap), representing a substantial operational cost and an emerging conservation challenge. Using large amounts of natural bait to trap commercial species is a common trend among trap fisheries. For example, to capture one kg of Norway lobsters (*Nephrops norvegicus*), 1.1 kg of bait is used (Ungfors et al., 2013) and in the case of the American lobster (*Homarus americanus*) fishery, the bait to catch ratio can go as high as 1.9 kg of bait for every kg of catch (Harnish & Willison, 2009), making trap fisheries highly vulnerable to quota reductions or increases in bait price. In the Barents Sea snow crab fishery, the demand for bait is exacerbated by the number of traps used per vessel (12,000 trap maximum) (L. Olsen, 2018, personal communication) and the efficiency with which traps are deployed and hauled (∼1,000 traps per day).

Chemical attractants are released from the bait and transported downstream by the water current. The size and shape of the resulting odour plume strongly depends on the amount of bait, current speed, direction, and turbulence (Sainte-Marie & Hargrave, 1987; Chiasson et al., 1992; Moore et al., 1994; Winger & Walsh, 2011). Snow crab are attracted by the smell of the bait (McLeese, 1970; Mackie, 1973; Hancock, 1974) from down current, crawling toward the trap (Lapointe & Sainte-Marie, 1992; Chiasson et al., 1992; Vienneau, Paulin & Moriyasu, 1993), and eventually they find the trap, climb the exterior walls and enter through the top entrance (Winger & Walsh, 2011). Squid imported from South America is currently the most commonly used bait when targeting snow crab being more efficient than other natural baits (Grant & Hiscock, 2009), however, fishermen may use fish to bait traps; in Canada herring is used in some occasions due to its low price and local availability (Grant & Hiscock, 2009).

Forage fish are commonly used as natural bait in trap fisheries worldwide (Chanes-Miranda & Viana, 2000). However, using these natural resources for the sole purpose of catching more valuable species represents a growing conservation and societal issue. Key concerns include: the rising cost of bait, proper ecosystem function when forage fish are removed, consumption of fossil fuels and production of CO_2_ for capture, and the fact that much of this seafood is already food grade quality suitable for human consumption (Cury et al., 2000; Dale, Siikavuopio & Aas, 2007; Grant & Hiscock, 2009; Driscoll & Tyedmers, 2010; Smith et al., 2011; Archdale & Kawamura, 2011; Essington et al., 2015). In response to these challenges, a growing body number of studies and companies have focused on the development of alternative baits (see review by Løkkeborg et al., 2014).

Historically there is evidence that different types of marine mammals were used by fishermen to bait traps in commercial crab fisheries (Lescrauwaet & Gibbons, 1994). Several studies have attempted to use waste from fish processing industries to create alternative bait with some success (Mackie et al., 1980; Chanes-Miranda & Viana, 2000; Dale, Siikavuopio & Aas, 2007; Beecher & Romaire, 2010; Archdale & Kawamura, 2011). However, to our knowledge, there has been no systematic attempt to investigate the use of marine mammal by-products as bait in snow crab fisheries.

Harp seals are commercially harvested in the Greenland Sea, White Sea, and Barents Sea. These fisheries are well managed and regulated jointly by Russia and Norway, with a total allowable catch (TAC) of 36,090 animals for 2017 (where two pups balance one animal) (Joint Norwegian—Russian Fisheries Commission, 2017). Minke whale is the only whale species that is allowed to be harvested in Norway and this popular meat is usually found in fish restaurants (Institute of Marine Research, 2013). Quotas set by the government are based on abundance estimates ratified by the Scientific Committee of the International Whaling Commission, ensuring a sustainable fishery that follows scientific advice and an ecosystem-based approach (Institute of Marine Research, 2017). The quota for 2017 was set to 999 whales, which is less than 1% of total abundance estimates (Institute of Marine Research, 2017).

This study evaluated the performance, in terms of catch rates, of locally available alternative baits manufactured from harp seal (*Pagophilus groenlandicus*) and minke whale (*Balaenoptera acutorostrata*) by-products in order to reduce the ecological impact and improve the efficiency of this bait intensive snow crab fishery.

## Materials and Methods

### Vessel and study area

Snow crab (*C. opilio*) was the species of interest in this study. The fishing trials were performed aboard a commercial snow crab fishing vessel in the Barents Sea, Norway during May 24th–June 24th, 2016. The vessel was 58 m long and had Gross Tonnage of 800 tonnes, and engine power of 3,000 hp (1350 AUX, 1650 Main). A crew of 18 worked onboard the vessel. Traps were emptied onto a sorting table. Legal-sized crabs (carapace width (CW) ≥ 100 mm) were transferred into a water-filled holding tank below deck using a slide and undersized crabs were returned to the sea. Afterward, snow crabs were processed in the onboard factory, cooked and frozen clusters were produced for immediate export.

**Figure 1:**
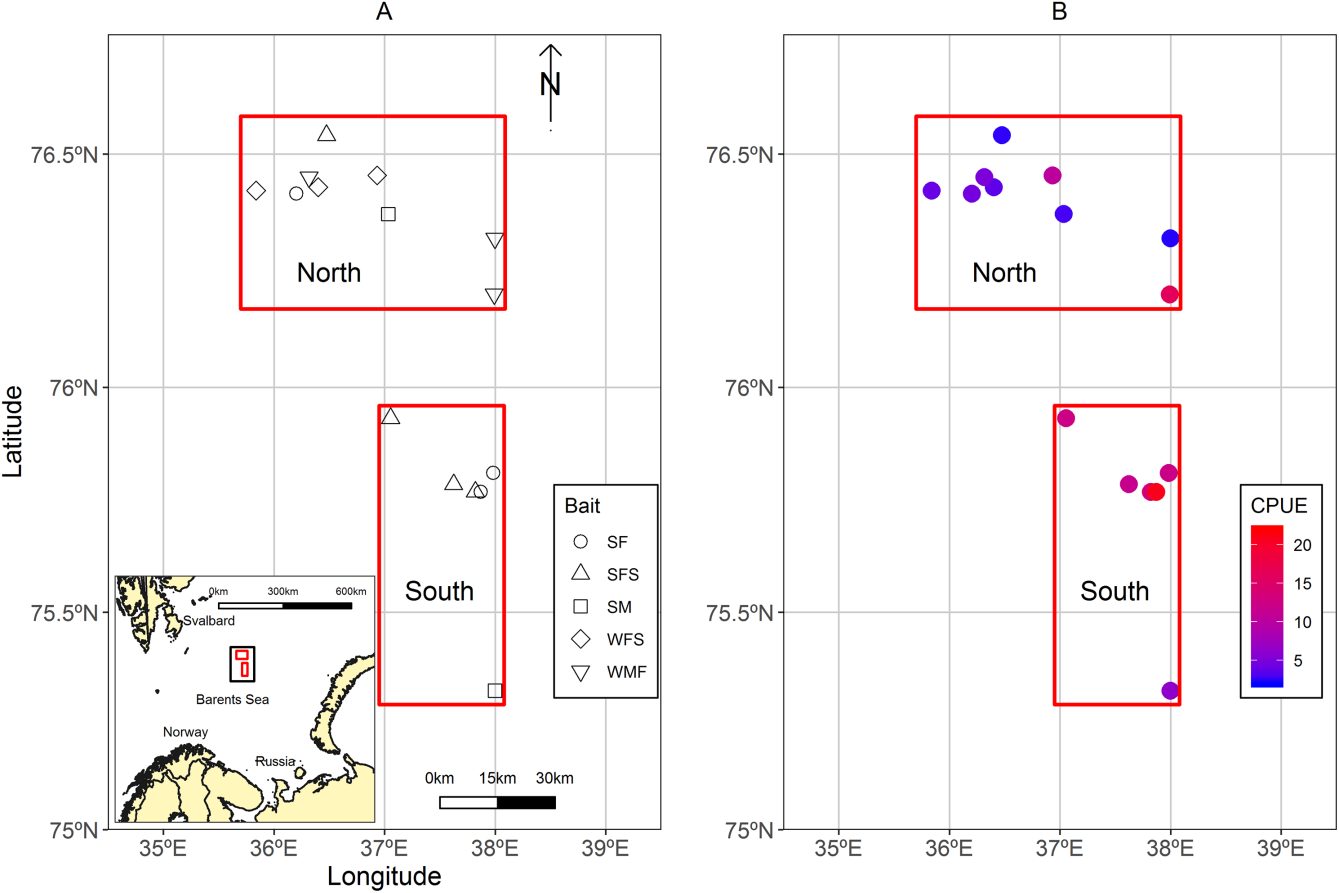
Maps of the study area located in the Sentralbanken area of the Barents Sea. (A) Map of the study area located in the Sentralbanken area of the Barents Sea with inset map of the broader area. Black rectangle in the inset map indicates the study area. Red rectangles indicate north and south locations. Shapes indicate positions off the fleets deployed and type of experimental bait used in the fleet. (B) Mean CPUE for the control traps in the different fleets. Map data from GADM database of Global Administrative Areas (http://gadm.org/). Mercator projection WGS 84 was used.

A total of 13 strings of traps (similar to a longline system), usually known as fleets were deployed in the Sentralbanken area of the Barents Sea (Fig. 1A). Fleets were grouped in north and south locations in relation to their proximity to each other and total average catch per unit effort (CPUE) of control traps (Fig. 1B). The water depths ranged between 210 and 288 m for the fleets.

### Fishing experiment

**Figure 2:**
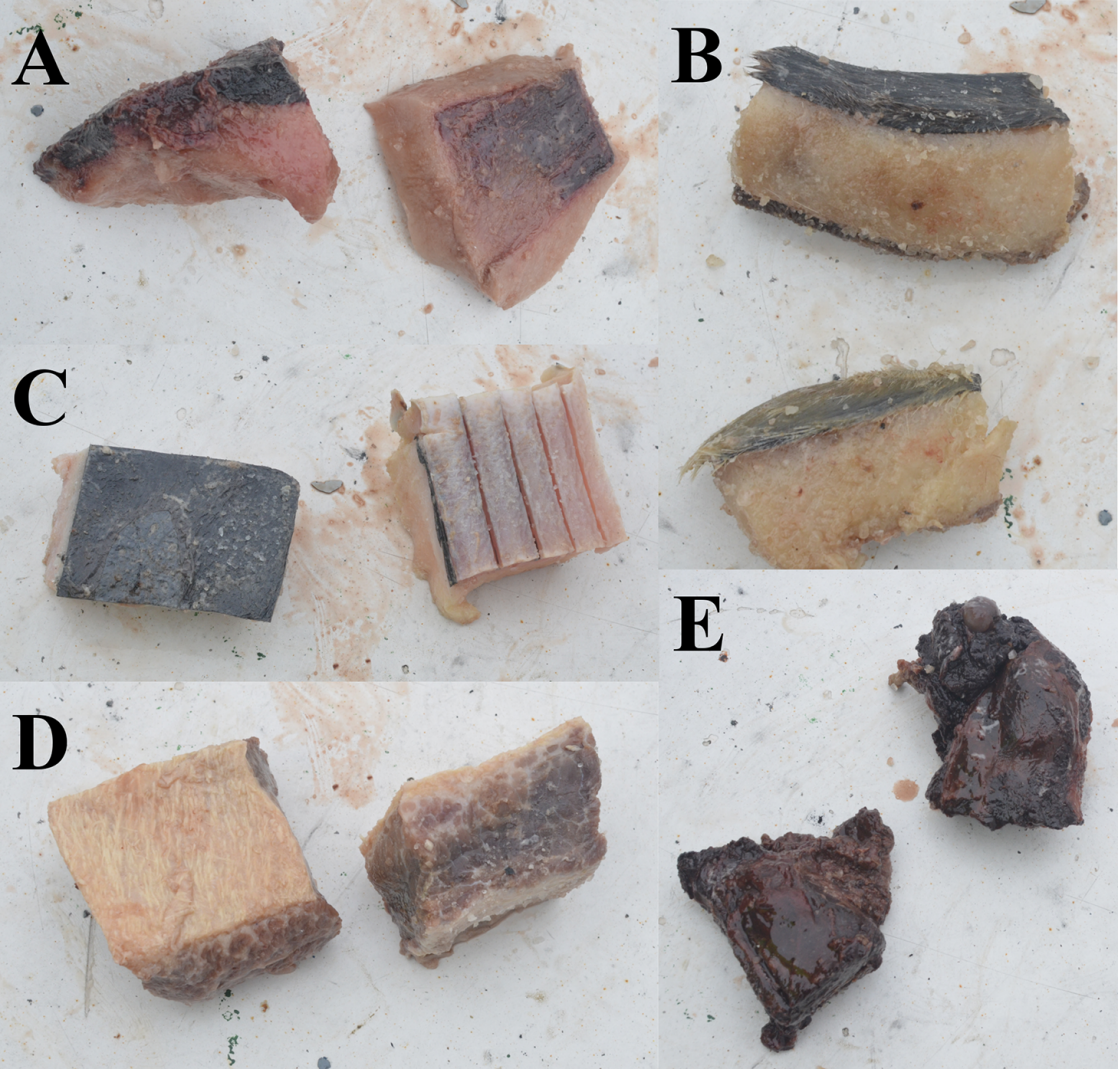
Alternative baits used in the experiment. (A) Seal fat. (B) Seal fat with skin. (C) Whale fat with skin. (D) Whale meat with fat. (E) Seal meat with bone.

**Figure 3:**
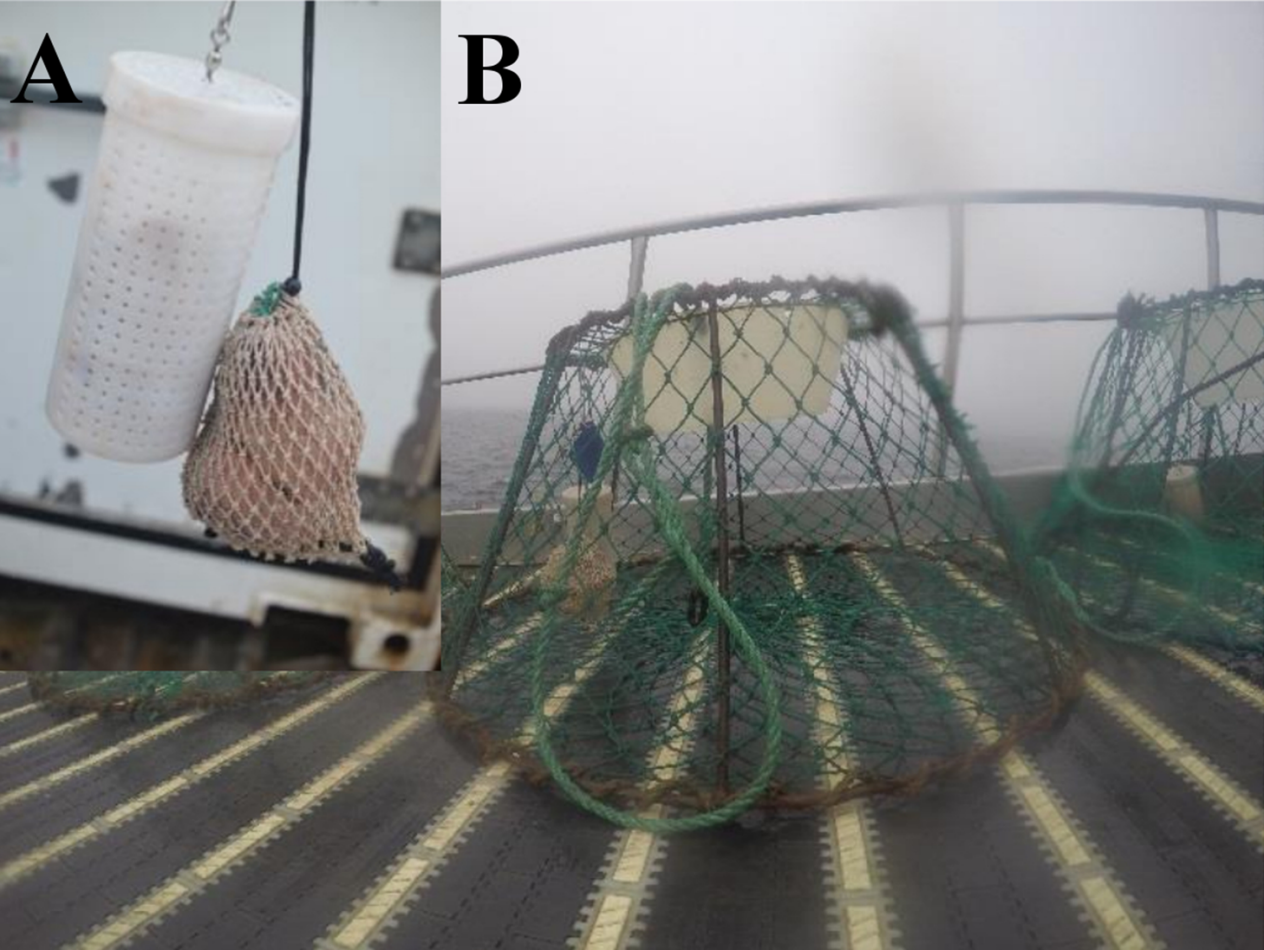
Japanese-style conical snow crab trap baited with seal fat in the mesh bag and plastic jar protection devices onboard of a Norwegian snow crab fishing vessel. (A) Closer look to the bait protection devices. (B) Snow crab pot.

Five different types of new alternative baits were evaluated; harp seal fat (SF), harp seal fat with skin (SFS), harp seal meat with bone (SMB), minke whale fat with skin (WFS), and minke whale meat with fat (WMF) (Fig. 2). Each bait was separately and randomly distributed within commercial fleets of traps which were baited with whole squid. Randomization of experimental traps in the commercial fleets was generated with excel software. Trials were conducted under commercial fishing conditions with the gear deployed and retrieved in the manner typical for this fishery. Traps were deployed in fleets ranging from 126 up to 197 traps spaced at intervals of 30 m. To avoid the probability that traps on the edges of a fleet may perform different from the rest of the traps, due to the floating line lifting these pots, the first and last five traps from the fleets were excluded from the results and subsequent analyses. All traps were small Japanese-style conical traps similar to those used in eastern Canada (see Winger & Walsh, 2011) with 140 mm stretched mesh, a top plastic entrance cone, a bottom ring diameter of 133 cm, and a volume of 2.1 m^3^ (Fig. 3). A total of 515 traps in 13 fleets were successfully deployed and hauled during the fishing trip (Table 1). Of these, 322 traps were baited with squid (i.e., control), 37 traps were baited with SF, 40 traps were baited with SFS, 19 traps were baited with SMB, 61 traps were baited with WFS, and 36 traps were baited with WMF (Table 2).

**Table 1:** Fleets of traps deployed during the experiment with their respective ID, type of experimental bait, number of experimental and control traps, geographic coordinates, soak time, and depth.

Fleet ID	Experimental bait	Experimental traps	Control traps	Latitude	Longitude	Soak time (days)	Depth (*m*)
45	Seal fat/skin	20	35	75°55′56″N	37°03′15″E	6.5	218
8	Seal fat/skin	20	32	75°46′12″N	37°49′12″E	11.3	210
10	Seal fat	17	34	75°46′12″N	37°52′6″E	11.3	212
38	Whale meat/fat	9	10	76°12′6″N	37°59′24″E	11.4	262
12	Seal meat/bone	10	20	75°19′18″N	37°59′48″E	11.5	220
35	Whale fat/skin	19	30	76°27′18″N	36°56′00″E	13.7	262
42	Seal fat	14	18	76°25′00″N	36°12′12″E	11.3	288
24	Whale meat/fat	9	18	76°27′06″N	36°19′00″E	11	220
19	Seal fat	6	10	75°48′42″N	37°58′54″E	5.3	220
13	Whale meat/fat	18	33	76°19′18″N	37°59′48″E	4.9	262
18	Seal meat/bone	9	10	76°22′24″N	37°02′00″E	4.7	270
1	Whale fat/skin	29	54	76°25′24″N	35°50′24″E	4.6	282
2	Whale fat/skin	13	16	76°25′48″N	36°24′6″E	4.5	266

**Table 2:** Mean CPUE ± SE of the different bait treatments with their respective sample size (*n*), mean soak time ± SE and mean depth ± SE.

Treatment	Mean CPUE	*n*	Mean soak time (days)	Mean depth (*m*)
Control	9.87 ± 0.45	322	8.5 ± 0.19	245 ± 1.63
Seal fat	13.84 ± 2.12	37	10.3 ± 0.37	242 ± 5.99
Seal fat/skin	13.00 ± 0.85	40	8.9 ± 0.38	214 ± 0.64
Seal meat/bone	2.05 ± 0.46	19	8.3 ± 0.80	244 ± 5.88
Whale fat/skin	1.79 ± 0.49	61	7.4 ± 0.55	272 ± 1.20
Whale meat/fat	2.81 ± 0.70	36	8.1 ± 0.53	252 ± 3.07

Shielding of the bait from predation by non-target species was accomplished by placing the bait in perforated plastic jars and mesh bags (Fig. 3). Each trap was baited using one jar and one mesh bag of bait, which were hung together in the center of the trap attached to the top mesh of the trap. Control traps were baited with one kg of squid; 0.5 kg in the bag and 0.5 kg in the jar following the actual bait configuration used during commercial fishing operations. Experimental traps were baited with the same amount of alternative bait, which was cut in pieces approximately 0.17 kg each in order to mimic the number of pieces used in the control traps (e.g., six pieces of new bait: six whole squids: one kg of bait). Alternative baits were locally obtained from sealers and whalers in Tromsø, Norway. Bait costs and quantity used during the fishing trip were recorded in order to produce a simple bait cost analysis. Squid bait was thawed before baiting the traps, whereas, the alternative baits did not require thawing as they were preserved in barrels with salt and water. When hauling the traps the bait remaining in the perforated plastics jars and mesh bag was carefully observed in order to qualitatively assess bait depletion.

The time during which the traps were in the ocean (soak time), depth, and position (latitude and longitude) were recorded for all deployments. Due to the large numbers of traps in each fleet, only traps situated either side of the experimental traps, were declared control traps. All other traps were considered commercial gear and were not included in this analysis. Randomization of the distribution of experimental traps produced a consecutive or intercalated position of the experimental traps in determined sections of the fleet, thus, the number of control traps was less than double when compared to the number of experimental traps. CW was measured for randomly selected crabs, including sublegal individuals, females and soft-shelled crabs if present in the random sample. Once the trap was emptied on to the sorting table, the crab located nearest to the researcher was measured. If time permitted, additional crab were measured. It was possible to measure one to three individuals per trap depending on the available time before the next trap arrived. It was not possible to measure CW for all crabs due to the constant hauling of the fishing gear, processing of the crabs, and limited workspace onboard the vessel. Once CW was measured, if under-sized, soft shelled and female crabs were present, they were rapidly returned to the sea by the crewmembers, in order to be able to rapidly record the number of male legal-sized, hard-shell snow crab (≥100 mm carapace width) per trap hauled (CPUE) before the next trap arrived. Limited time between trap arrivals constrained our ability to count non-commercial crabs and attempting to do this would have slowed down the hauling of traps, representing an economic loss for the fishing enterprise.

### Statistical analysis

Catch per unit effort was treated as count data and was not transformed to satisfy normal distribution of the data (O’Hara & Kotze, 2010). CPUE was modeled using a generalized linear model (GLM) framework. A Poisson GLM with a log link function was attempted, but showed overdispersion, shifting analysis to a negative binomial GLM. The model was fit using the glm.nb function of the MASS package (Venables & Ripley, 2002) in R statistical software (R Development Core Team, 2009). We fit the model

$${\rm log}\left( y \right) = \; {\rm \alpha } + \; {{\rm \beta }_1}{\rm bait} + {{\rm \beta }_2}{\rm Soak\; time}*{{\rm \beta }_3}{\rm Location}\,{\rm + }\,{\rm \varepsilon }$$

where *y* is CPUE, α is the intercept, β_1_bait is bait treatment, and β_2_Soak time * β_2_Location is the interaction term which considers the interaction between trap soak time and trap location (north or south), and ε is the error term.

Snow crab CW was compared among the different treatments with a Gaussian generalized linear mixed-effects models (GLMMs; Zuur et al., 2009) using R statistical software with the glmmTMB function (Brooks et al., 2017). We fit the model

$$y = \; {\rm \alpha } + \; {{\rm \beta }_1}{\rm bait} + b + {\rm \varepsilon }$$

where *y* is CW, α is the intercept, β_1_bait is the bait treatment, *b* is the random variable representing the variability among fleets (where *b* ∼ *N* (0, σ^2^)), and ε is the error term.

Size-based selectivity ratios could unfortunately not be computed as our sampling technique did not document the proportion of individuals measured for CW.

Generalized linear model and GLMM models were tested for outliers, over/under-dispersion, independence, homogeneity, and normality (as appropriate for each model) according to the techniques described in Zuur, Ieno & Elphick (2010) and Zuur & Ieno (2016). Residuals were compared against fitted values. A *p*-value of <0.05 was considered to indicate statistical significance. Significance of model variables was checked with a likelihood-ratio test using drop1 function of the drop1.merMod package in R (Bates et al., 2015) (following Zuur et al., 2009 procedures). When terms were found to be non-significant, the highest *p*-value term was removed and the model was refitted until all terms were significant. Final models are shown in the results section with their respective parameter estimates, standard errors, *Z*-values, and *p*-values.

Multiple non-parametric Two-sample Kolmogorov–Smirnov tests were used to check the null hypothesis that control traps CW cumulative distribution is the same with the cumulative distribution of CW for each of the bait treatments. These comparisons were performed separately for north and south locations. A *p*-value of <0.05 was considered to indicate statistical significance.

## Results

### CPUE in relation to bait type, soak time and location

**Figure 4:**
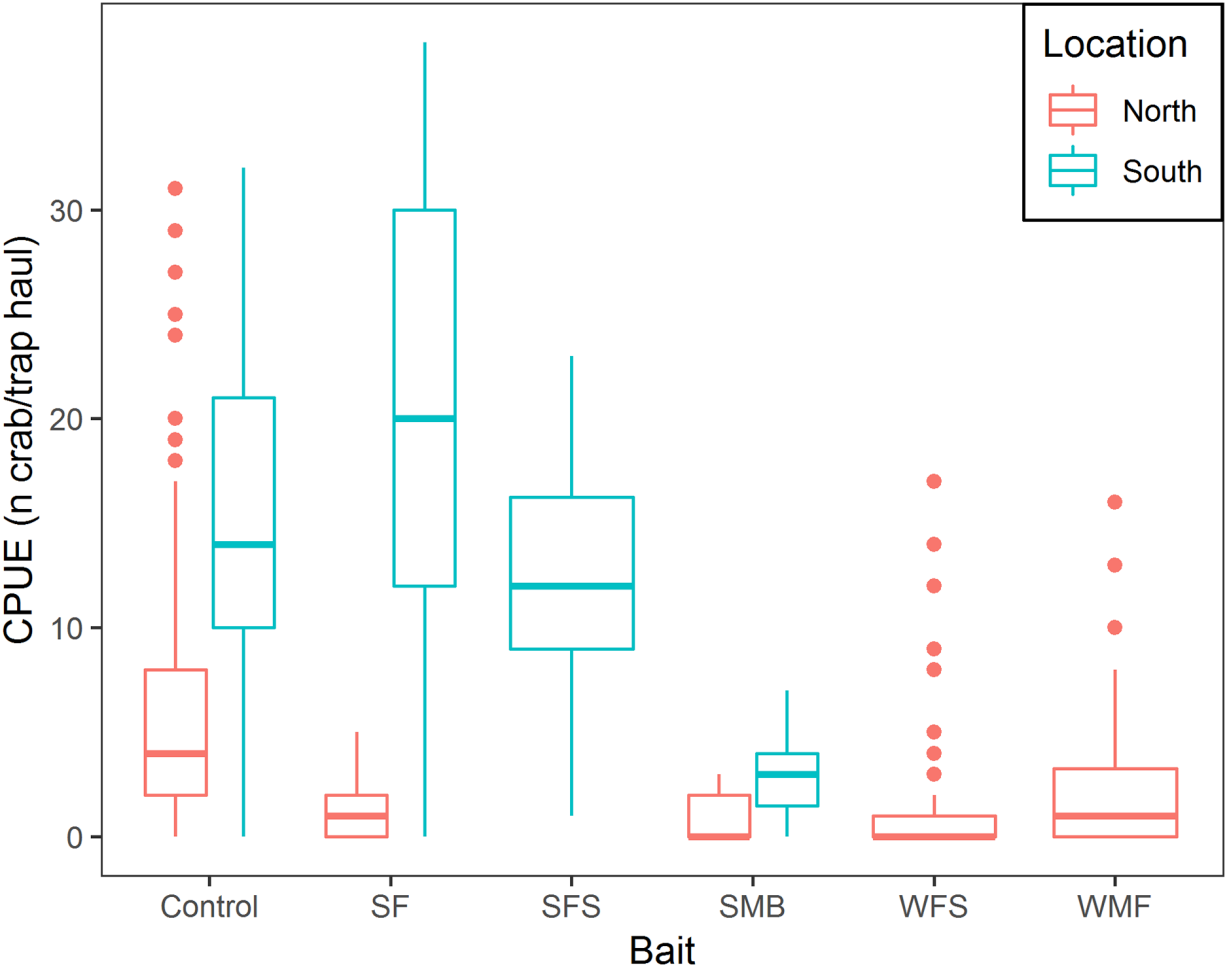
Boxplot of CPUE of snow crab for the different bait treatments in north and south locations. Colors represent north and south locations. Horizontal line in the middle of the boxes represent the median CPUE. Lower and upper limit of the boxes show the first and third quartile, respectively. Lower and upper whiskers represent scores outside the interquartile range. Dots represent the outliers.

Mean CPUE, sample size, mean soak time, and mean depth are shown in Table 2. SF and SFS produced a CPUE that was not statistically different from the traditional bait (*p*-value = 0.325 and 0.069, respectively, Table 3). Mean CPUE for the remaining baits were statistically lower than the traditional bait (*p*-values ≤ 0.05, Table 3). Overall, control traps produced a median of 8.0 crabs per trap haul (Fig. 4); traps baited with SMB captured 79.3% fewer crabs (95% CI [87.1–67.1]), WFS captured 75.8% fewer crabs (95% CI [81.9–67.8]), and WMF 57.7% fewer crabs (95% CI [69.1–42.2]), according to model estimates (Table 3; Fig. 4).

**Table 3:** Parameter estimates from the Negative Binomial GLM.

	Estimate	Standard error	*Z*-value	*P*-value
Intercept	0.354	0.121	2.931	0.003
SF	−0.120	0.122	−0.984	0.325
SFS	−0.224	0.123	−1.816	0.0694
SMB	−1.577	0.234	−6.747	<0.001
WFS	−1.419	0.151	−9.383	<0.001
WMF	−0.860	0.162	−5.306	0.001
Soak time	0.162	0.013	12.924	<0.001
Location: South	2.257	0.233	9.700	<0.001
Soak time * Location: South	−0.142	0.023	−6.066	<0.001

**Figure 5:**
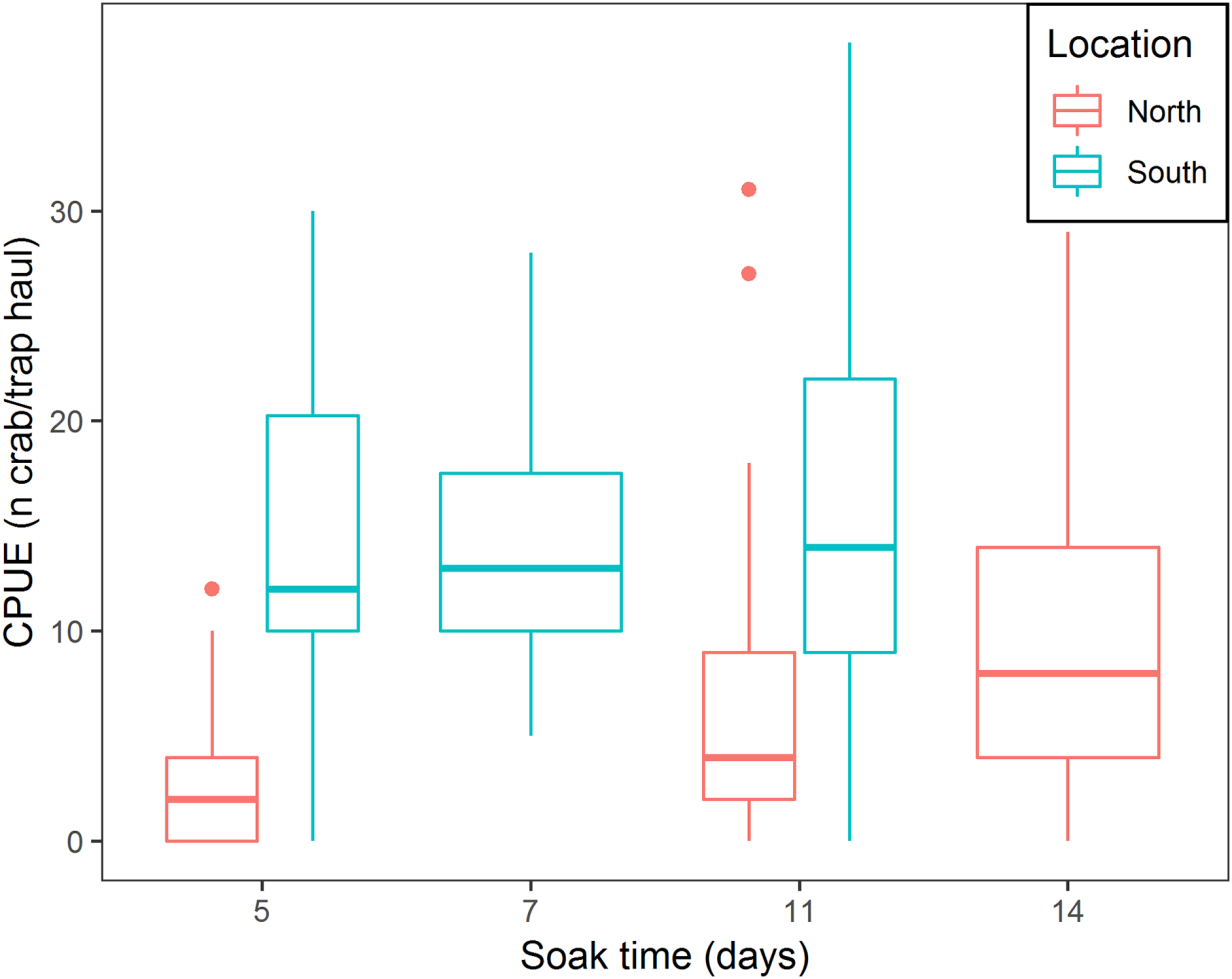
Boxplot of CPUE for all bait treatments combined for north and south locations across the different soak times. Colors represent north and south locations. Horizontal line in the middle of the boxes represent the median CPUE. Lower and upper limit of the boxes show the first and third quartile, respectively. Lower and upper whiskers represent scores outside the interquartile range. Dots represent the outliers.

There was a positive relationship between CPUE and soak time; every additional day of soak time increased, on average, CPUE by 17.6% (95% CI [14.8–20.6]) (Table 3; Fig. 5). For traps located in the north, a median of 3.0 crabs per trap in total was observed, while fleets in the south captured, on average, 9.6 times more crabs per trap (95% CI [6.1–15.1]) (Table 3; Fig. 5). In the south location, for every additional day of soak time, we observed 0.87 fewer units of increase in CPUE, compared to north location (95% CI [0.83–0.91]) (Table 3; Fig. 5).

### CW comparison for the different bait treatments

**Figure 6:**
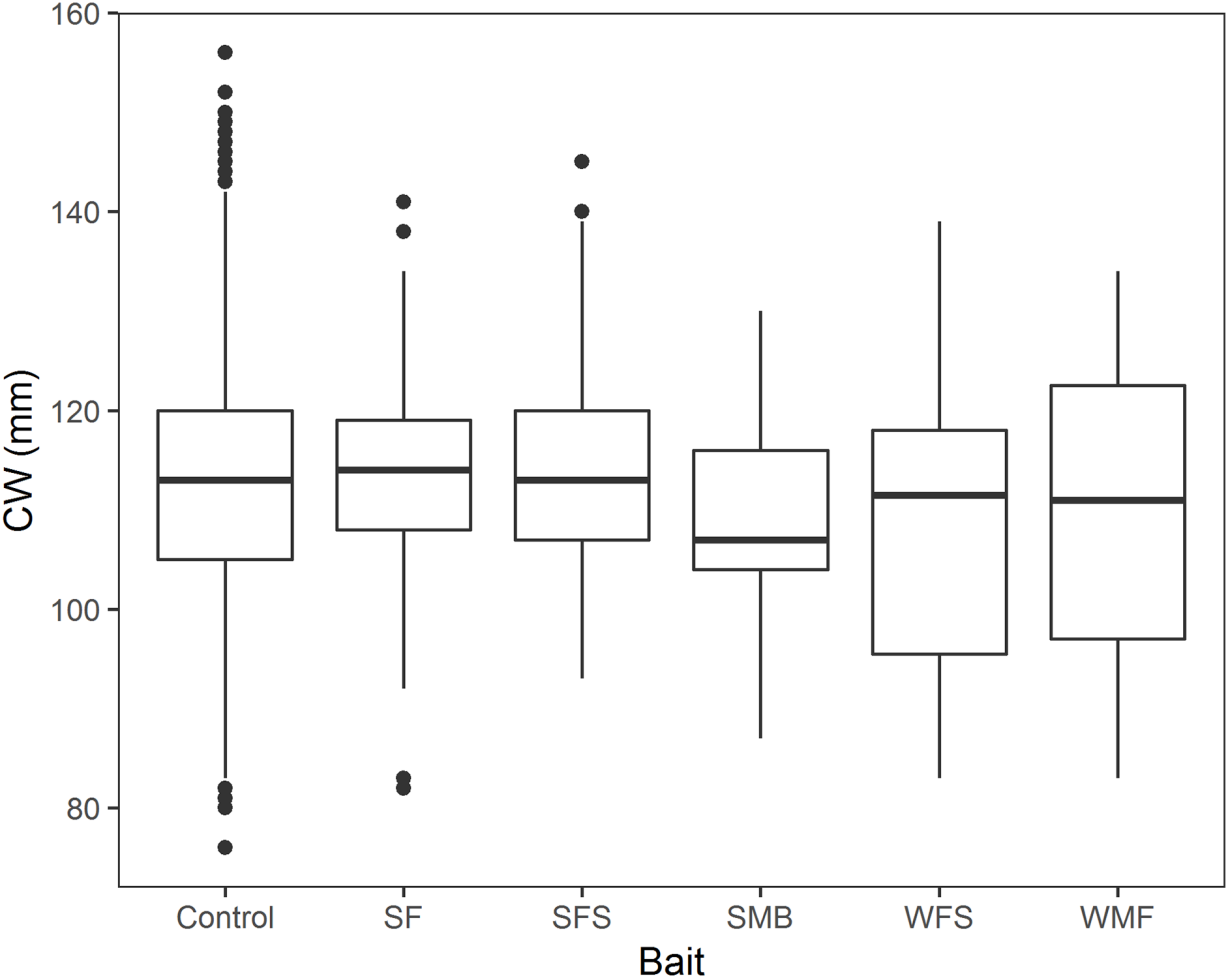
Boxplot of CW of snow crab for the bait treatments from north and south locations combined. Horizontal black line in the middle of the boxes represent the median CPUE. Lower and upper limit of the boxes show the first and third quartile, respectively. Lower and upper whiskers represent scores outside the interquartile range. Black dots represent the outliers.

A total of 2,838 crabs were measured in the 13 fleets of traps. Mean CW, sample size and percentage of sublegal sized crabs are shown in Table 4. Female and soft-shelled crab were not present in the randomly measured crabs. We found no significant effect of bait treatment on snow crab CW (*p*-values > 0.05, Table 5; Fig. 6). Model estimates showed no statistical relationship of CW with SF (β_1_ = 2.231, 95% CI [−1.434–5.896]), SFS (β_2_ = 0.786, 95% CI [−2.145–3.716]), SMB (β_3_ = −3.046, 95% CI [−9.352–3.267]), WFS (β_4_ = −4.593, 95% CI [−9.435–0.248]), or WMF (β_5_ = −2.941, 95% CI [−7.611–1.729]), when compared to control traps (Table 5).

**Table 4:** Mean CW ± SE of the different bait treatments with their respective sample size (*n*) and percentage of sublegal-sized crabs.

Treatment	Mean CW (mm)	*n*	Sublegal-sized crab (<100 mm) (%)
Control	112.75 ± 0.26	2,601	16.03
Seal fat	113.49 ± 1.69	53	11.32
Seal fat/skin	113.47 ± 1.06	106	8.49
Seal meat/bone	108.82 ± 3.10	17	17.65
Whale fat/skin	109.50 ± 2.70	30	26.67
Whale meat/fat	109.94 ± 2.63	31	29.03

**Table 5:** Parameter estimates from Gaussian generalized linear mixed-effects model.

	Estimate	Standard error	*Z*-value	*P*-value
Intercept	112.640	0.740	152.230	<0.001
SF	2.231	1.870	1.190	0.233
SFS	0.786	1.495	0.530	0.599
SMB	−3.043	3.219	−0.950	0.345
WFS	−4.593	2.470	−1.860	0.063
WMF	−2.941	2.383	−1.230	0.217

**Figure 7:**
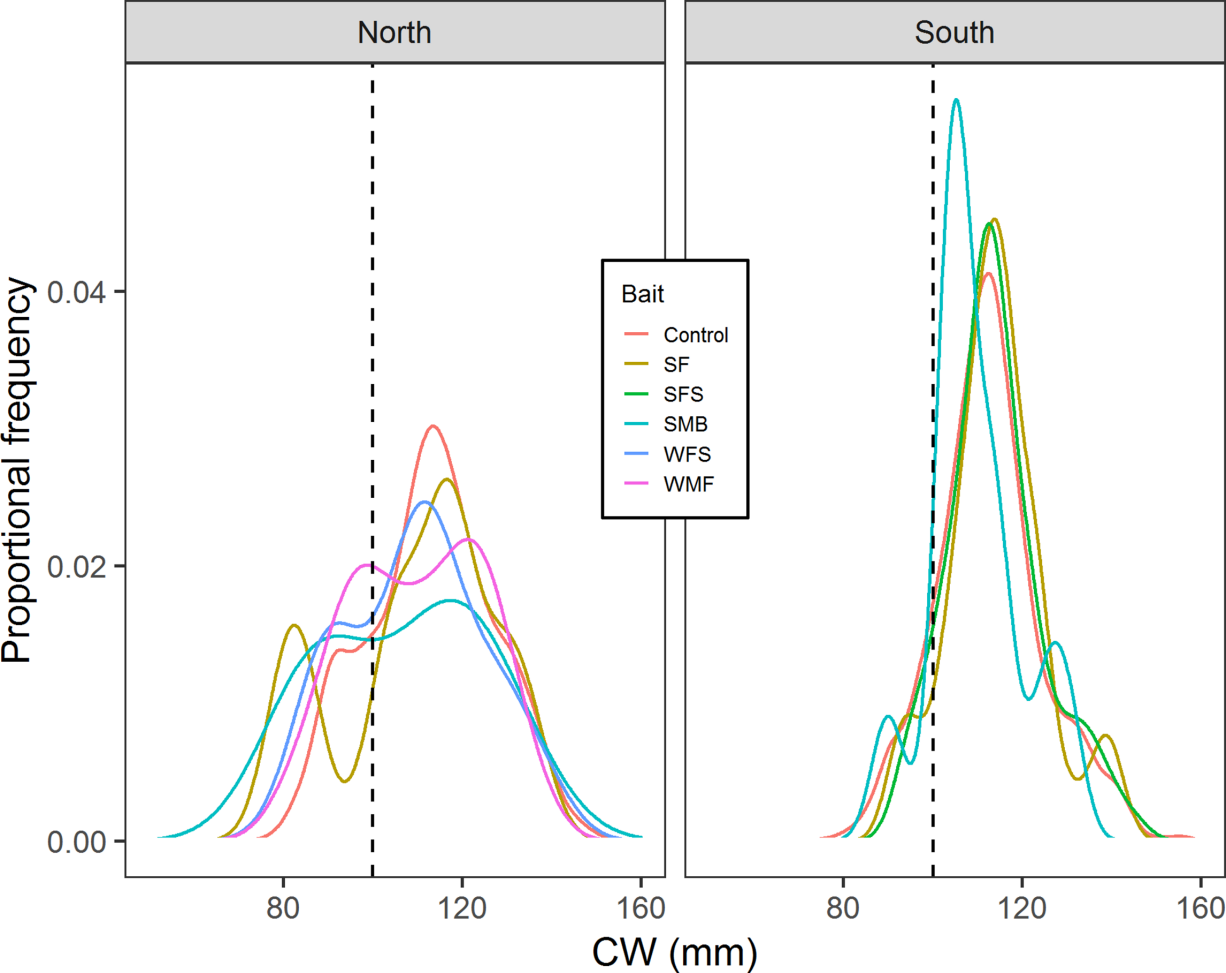
Proportional frequency distribution of CW (mm) for the different bait treatments in north and south locations. Dashed back lines show the limit between sublegal and legal-sized crabs (100 mm).

Results from multiple Two-sample Kolmogorov–Smirnov tests suggested that in the north location cumulative distribution of CW from control traps is the same with the cumulative distributions of CW from SF (*D* = 0.212, *p*-value = 0.814), SMB (*D* = 0.355, *p*-value = 0.556), WMF (*D* = 0.142, *p*-value = 0.575), and WFS traps (*D* = 0.270, *p*-value = 0.435) (Fig. 7). Results from south location indicated that SF (*D* = 0.109, *p*-value = 0.693), SFS (*D* = 0.0740, *p*-value = 0.662), and SMB (*D* = 0.270, *p*-value = 0.354) traps had the same cumulative distribution of CW when compared to control traps (Fig. 7).

### Bait purchase price comparison

During the fishing trip, 23,000 kg of whole squid were used to bait the traps. SF was 57% cheaper than traditional bait and purchased from a provider that sells this by-product for oil production. SFS was bought from two different providers, at 35% cheaper and 8% more expensive compared to traditional bait. SMB price can vary depending on the availability, increasing bait costs by 201–545%, while, WMF and WFS have no commercial value (Table 6). A simple bait cost analysis, based on differences in bait prices during the fishing trip, suggests that this fishery could experience a minimum savings of $1.6 million CAD per fishing season (i.e., 1 year) when substituting squid by SF bait, this is, when each of the five vessels operating uses the maximum limit of 12,000 traps, hauling them every three weeks (at minimum according to regulations) for a period of 9 months (fishery closure from June 15th to September 15th).

**Table 6:** Bait price and total fishing trip bait cost in NOK and CAD (conversion rate June 20, 2018).

Bait	Price NOK/kg (CAD/kg)	Total cost per fishing trip: 23,000 kg NOK (CAD)	Cost reduction or increase
Squid	23.25 (3.78)	534,750 (86,940)	–
SF	10 (1.62)	230,000 (37,260)	−57%
SFS	15–25 (2.44–4.06)	345,000–575,000 (56,120–93,380)	−35% and +8%
SMB	70–150 (11.37–24.37)	1,610,000–3,450,000 (261,510–560,510)	+201% and +545%
WFS	No commercial value		
WMF	No commercial value		

### Qualitative observations of bait depletion

Qualitative observations from 515 trap hauls revealed that bait depletion was always higher for baits contained in mesh bags compared to bait contained in perforated jars. Control traps (i.e., squid bait) showed higher bait depletion compared to experimental traps (SF, SFS, SMB, WMF, and WFS); these suffered less depletion and greater amounts of remaining bait were observed in the protection devices. Depletion of bait was also observed in traps with no crabs.

## Discussion

### CPUE and CW

This study represents the first systematic attempt to investigate the performance of alternative baits derived from marine mammal by-products for the commercial capture of snow crab. Of the five alternative baits evaluated, SF and SFS showed no significant effect on CPUE (*p*-value = 0.325 and 0.069, respectively), indicating that SF and SFS produced catch rates comparable to the traditional squid bait. All of the other experimental baits (SM, WFS, and WMF) significantly decreased CPUE, when compared to squid. No significant effect of bait treatment on CW was detected and the cumulative distribution of CW was the same when comparing control traps to SF, SMB, WMF, and WFS traps in north location, as well as the same when comparing control traps to SF, SFS, and SMB baits in south location. Overall our results indicated that none of the baits produced significantly different CW or CW cumulative distribution, when compared to control traps. These results showed that SF and SFS baits not only performed as well as squid in terms of number of commercial crabs per trap, but they also captured the same size and size cumulative distribution when compared to squid.

Whole squid contains an abundance of proteins, lipids, and minerals (Lian, Lee & Park, 2005) which effectively attract snow crabs into the trap. Several studies have shown that amino acids and related compounds are feeding stimulants for crustaceans (McLeese, 1970; Kay, 1971; Mackie, 1973; Hindley, 1975; Hartman & Hartman, 1977; Carr, Netherton & Milstead, 1984; Carter & Steele, 1982; Zimmer-Faust et al., 1984; Johnson & Atema, 1986; Kreider & Watts, 1998). We therefore expected that baits containing meat would produce higher catch rates, such as SMB and WMF, which had higher contents of amino acids. However, our results showed that baits containing fat form harp seal performed as well as squid. Both, whole squid (Lian, Lee & Park, 2005) and SF (Brunborg et al., 2006) have high contents of fatty acids, which contain water-soluble organic molecules that can act as chemical attractants for crustaceans (Westerberg & Westerberg, 2011). Precise explanations for why snow crab preferred SF and SFS are uncertain, however, a common denominator with squid appears to be the presence of lipids (i.e., fatty acids). Furthermore, it was proven that experimental baits that did not contain SF did not perform as well, suggesting that the fat of the seal has attractive properties. Further investigation is warranted to exactly characterize what compounds present in the SF determine its effectiveness.

This experiment had several limitations due to the commercial nature of the fishing vessel. Sample sizes of the experimental traps were lower when compared to the control traps in both, CPUE and CW data. This unbalanced nature of the experiment could have lead to a general loss of statistical power, increasing the likelihood of a type II error. This means that if the sample size of experimental traps were to be increased we could have potentially found significant differences between control and SF baits (in CPUE and CW), possibly indicating that these baits could have performed better than squid in terms of catch rates and size of the crabs.

Counts of commercial crabs and measurement of one to three crabs per trap provided limited information about catch composition of the traps and its effects on catch rates. It is known that the catch rate of a trap is affected by the type (male, female, soft-shelled, and dead crabs) and size of the crabs that are occupying the trap in the first place. Sainte-Marie & Turcotte (2003) showed that occupancy of the trap by adult males, recently moulted males, or dead crabs in first place reduced catch rates of the gear. According to Miller (1978) crabs approaching the trap are intimidated by the ones inside of it. Dead conspecifics inside the trap deter crabs from entering because they avoid death or wounds (Sainte-Marie & Turcotte, 2003). Count and measurement of all crabs would have provided important information that could have potentially further explained catch rates of the traps, other than soak time, location and bait treatment.

### Soak time and location

The results of this study showed that CPUE increased with longer soak time. These results align with recent findings by Nguyen et al. (2017) who documented increasing CPUE for soak times up to 195 h. This experiment showed that increasing soak time increased CPUE (17.6% on average per day), however, the effect varied between the north and south locations. In the south location, where catch rates were higher, CPUE increased at a lower rate with increasing soak time (for every additional day of soak time, we observed 0.87 fewer units of increase in CPUE), while in the north location higher rates of increase in CPUE with increasing soak time were observed. We attribute this finding to spatial variation in snow crab abundance. In the south location, where higher abundance occurs, more crabs will enter the trap sooner, reaching trap saturation earlier, further reducing catchability of the trap (Miller, 1990), therefore CPUE will slowly increase with increasing soak time. Cyr & Sainte-Marie (1995) observed that bait quantity produced a similar effect on CPUE increments; increasing bait quantity attracted more crabs to the trap initially, increasing trap saturation and producing lower rates of increase in CPUE. In the north location, low abundance of crabs allows a gradual arrival of crabs to the trap over time, therefore, every additional day will increase CPUE rates to a greater extent.

### Success of a new bait

In addition to catchability, the success of any new bait in a commercial fishery depends on its availability, storage logistics, and price (Dale, Siikavuopio & Aas, 2007).

During 2017, a total of 2,000 harp seals were harvested in Norway, representing only 5% of the TAC (Joint Norwegian—Russian Fisheries Commission, 2017), which is certainly not enough to supply the demand of bait of approximately 770 tonnes for the five snow crab fishing vessels currently operating. Even in a scenario where all the TAC of harp seal is caught, the resulting fat produced, which represents 29% of the animal weight (Shahidi, 1998), would not be sufficient to fulfill the bait requirements of the Norwegian snow crab fishery. SF from other regions (e.g., Newfoundland and Labrador, Canada) could represent a solution to fulfill the bait requirements. Other solutions such as the one proposed by Archdale & Kawamura (2011), where 30.7% of the bait comes from fish processing waste and the rest is a compound consisting of components such as wheat starch, garlic and brown sugar, which have been found to attract swimming crabs (Archdale & Nakamura, 1992; Kawamura et al., 1995; Archdale & Kawamura, 2011), reducing the amount of natural bait required. It is also recommended to test the performance of other by-products from land farming, these may represent another locally available option of an alternative bait. For example, Middleton et al. (2000) proved that a bait formulated from poultry mortality could be used as an alternative bait to harvest blue crabs.

Alternative baits used in these experiments have the advantage that they can be preserved in barrels with salt and water, and they do not require long-term freezer storage, as squid does. A comparison of the purchase prices illustrates that SF bait is cheaper than squid by 57%, and SFS can vary between 35% cheaper and 8% more expensive compared to squid, and squid is the preferred bait type at the present time. A simple bait cost analysis indicates that this fishery could experience a minimum savings of $1.6 million CAD if SF is used as a substitute for traditional squid bait. Given the recent decrease in Norwegian snow crab landings, this bait could present an opportunity for significant operational cost savings. For this study, the small amount of SF and SFS bait was sourced from a value chain that produces seal oil. It is conceivable that an even better price could be negotiated if larger amounts were purchased, thereby lowering the price even further. All of these attributes potentially offer significant opportunity to reduce the operational costs for fishing enterprises.

There are additional conservation benefits that favor the implementation of SF and SFS baits in this fishery. Firstly, the use of seal by-products would reduce the dependence on food grade squid that is already suitable for human consumption (Dale, Siikavuopio & Aas, 2007), especially with the growing demand for fish products and the importance of fish in human nutrition and food security (FAO, 2018). Secondly, the usage of locally available SF produces a bait with reduced fuel consumption, when compared to the elevated fuel consumption required to harvest forage fish (i.e., squid bait) (Driscoll & Tyedmers, 2010) and ship the bait (i.e., squid from South America), contributing to an activity with lower carbon footprint. And thirdly, utilizing seal by-products as bait it is also ecologically beneficial in the sense that reduces waste and contributes to the full usage of the animal. Similar to this is the utilization of salmon heads and bones to catch lobster, crab, and *Nephrops* in Scottish waters, increasing the economic benefits of salmon producers and minimizing waste sent to landfills (Murray, 2015).

### Bait depletion and quantity

Depending on the location and time of year, baits that are exposed in traps may be depleted by scavenging species within a few hours of deployment, losing their attractant properties and decreasing catch rates (Richards & Cobb, 1987; Robertson, 1989; Miller, 1990). To avoid this unwanted depletion, fishing enterprises tend to use bait protection devices (i.e., shields). In this study, qualitative observations indicated that baits contained in mesh bags were, in all traps, more depleted than baits contained in jars. Depletion of bait was also observed in traps with no crabs, indicating the presence of animals, other than snow crab, that feed from the bait (Dale, Siikavuopio & Aas, 2007) and exit the trap. Although not quantified, it was noted that all of the experimental baits experienced less depletion compared to squid. In fact in most of the cases there were high quantities of experimental bait remaining in the bait shields. This suggests that lower quantities of SF or seal fat and skin may be used, or that traps can be soaked for longer periods of time, or that bait may be re-used in subsequent trap deployments, all of which are potential means to reduce operational costs and address the issue of SF and SFS baits availability. Further studies on these matters are recommended.

Several studies have shown that the catchability of decapod crabs increases with increasing bait quantity (Thomas, 1954; Zimmer-Faust & Case, 1982, 1983; Miller, 1983; Takeuchi, 1988; Cyr & Sainte-Marie, 1995). This variable was not manipulated in this study, but could prove to be a valuable hypothesis for further evaluation. For example, it would be interesting to study the sensitivity of CPUE to varying amounts of the alternative baits presented here.

## Conclusions

In conclusion, this study evaluated the performance of several new alternative baits for catching snow crab in the Barents Sea. Each of the baits was developed from a waste stream (i.e., by-product) from seal and whale capture. Observations indicated that SF and SFS from harp seals were the best performing alternative baits, producing catch rates comparable to squid which is the current preferred bait type by industry, with no differences in size or size cumulative distribution of the crabs. Addressing the availability issue of SF to further implement this alternative bait, would reduce operational costs, contribute to a more environmentally friendly fishing activity, with a locally available lower carbon footprint bait that is not based in products suitable for direct human consumption.

## Supplemental Information

10.7717/peerj.6874/supp-1 Supplemental Information 1 Raw catch data. Raw data of the number of crabs caught per trap, including pot number, line/fleet number, bait treatment, depth, set and haul date.

10.7717/peerj.6874/supp-2 Supplemental Information 2 Raw carapace width data. Raw data of snow crab carapace width, including line/fleet number, bait treatment, sex, depth, set and haul date.

10.7717/peerj.6874/supp-3 Supplemental Information 3 Fleet raw data. Raw data of the fleets, including fleet number, bait treatment, latitude, longitude, set and haul date.

## Additional Information and Declarations

### Data Availability

The following information was supplied regarding data availability: Raw data is available in Files S1, S2, and S3. File S1 shows the catch data in terms of number of crabs per trap (CPUE), trap number, fleet number, soak time, depth, and set/haul dates. File S2 shows carapace width (CW) measurements for the snow crabs in mm, snow crab sex, fleet number, soak time, set/haul date, depth. File S3 shows fleets information, such as: fleet number, set/haul date, soak time, bait type, latitude, and longitude.
